# Nutrition Labelling Use and Higher Adherence to Mediterranean Diet: Results from the DiSA-UMH Study

**DOI:** 10.3390/nu10040442

**Published:** 2018-04-03

**Authors:** Eva María Navarrete-Muñoz, Laura Torres-Collado, Desirée Valera-Gran, Sandra Gonzalez-Palacios, Laura María Compañ-Gabucio, Sergio Hernández-Sánchez, Manuela García-de-la-Hera

**Affiliations:** 1 Centro de Investigación Biomédica en Red de Epidemiología y Salud Pública (CIBERESP), 28029 Madrid, Spain; sandra.gonzalezp@umh.es (S.G.-P.); manoli@umh.es (M.G.-d.-l.-H.); 2 Unit of Nutritional Epidemiology, Universidad Miguel Hernandez, 03550 Alicante, Spain; l.torres@umh.es (L.T.-C.); dvalera@umh.es (D.V.-G.); lcompan@umh.es (L.M.C.-G.); 3 Alicante Institute for Health and Biomedical Research (ISABIAL-FISABIO Foundation), 03550 Alicante, Spain; 4 Occupational Therapy Area, Surgery and Pathology Department, Universidad Miguel Hernandez, 03550 Alicante, Spain; 5 Vicerrectorado de Relaciones Institucionales, Universidad Miguel Hernández, Programa UMH Saludable, 03202 Elche, Spain; sehesa@umh.es

**Keywords:** Mediterranean diet, nutrition labelling use, student, young adult

## Abstract

The aim of this study was to identify determinants of the nutrition labelling (NL) use and explore its association with the adherence to Mediterranean Diet (MD) in Spanish health university students. We performed a cross-sectional analysis of the baseline data from 1026 university students aged 17–35 years enrolled in the DiSA-UMH (Dieta, Salud y Antropometría-Universidad Miguel Hernández) cohort study. Students were asked about their NL use by the following question: “Do you usually read the nutrition labelling of packaged foods?” (No, Yes). Dietary intake was assessed using a validated food frequency questionnaire and the adherence to MD was measured by relative Mediterranean Diet score (rMED). Socio-demographic, lifestyle, and anthropometric variables were also collected. Multiple logistic regression models were applied for the analysis. Fifty-eight percent of the students were NL users and were most likely to be women (Odds Ratio (OR): 1.38; 95% Confidence Interval (CI): 1.01–1.89), be older (OR per year: 1.08; 95% CI: 1.03–1.13), be physically active/very active (OR: 1.68; 95% CI: 1.17–2.41), and spend less time watching television (OR per hour: 0.84; 95% CI: 0.74–0.95). After adjusting for potential confounding factors, our findings suggested that those university students who had higher adherence to MD used NL greatly (OR per 2 points increase: 1.30; 95% CI: 1.18–1.43) and had a larger consumption of fish (OR per 100 g/day: 1.94; 95 CI: 1.38–2.71), vegetables (OR per 100 g/day: 1.15; CI 95%: 1.08–1.12), and fruits (OR per 100 g/day: 1.22; 1.11–1.34) and a smaller intake of meats (OR per 100 g/day: 0.76; 95% CI: 0.58–0.99). Our approach contributes to exploring the role of NL use as a suitable tool to make healthier food choices from a different wider perspective based on dietary patterns such as MD, which can also indicate an overall healthy lifestyle. Given the lack of research in Mediterranean areas, further studies focused on exploring the potential role of NL in promoting healthy dietary habits are required.

## 1. Introduction

Nutrition labelling (NL) is an important communication channel whereby consumers can obtain information about the nutritional content of packaged foods at the time of purchase, including the date of manufacture, storage conditions, cooking instructions, as well as the last date a food should be consumed. The health implications of NL are becoming a relevant issue in recent years because of its potential utility to promote healthier eating choices, making people more aware of their dietary practices [1,2,3]. A systematic review of 120 studies conducted in 2011 [4] showed that the prevalence of NL use in general population was above 50%, although variations across studies (82% in New Zealand, 52% in Canada, 47% in the EU, and 75% in the USA) were fairly evident. It should be noted that NL use can be influenced by individual characteristics (age, sex, and education); situational, behavioral, and attitudinal factors (working status, income, time spent shopping, etc.); product class involvement factors (importance of price, taste, ease of preparation, etc.); and nutrition knowledge [5]. Previous research has revealed that NL use was more prevalent among the female population, younger or middle-aged people, people on high incomes or with high education level, and those with healthier eating habits [2,4,6,7,8].

Regarding dietary practices, there is scant evidence from studies focused on the association between NL use and nutrients or foods, although the main findings suggest that a greater consumption of fruits, vegetables, fiber, calcium, potassium, vitamin D, and a lower intake of fat and sugar were associated with a higher NL use [9,10,11]. Parallel to the study of specific foods and nutrients, some researchers have explored the effects of NL use on the global diet, adopting an overall approach that allows the assessment of the existing possible interaction(s) with other compounds present in the food. In this regard, several studies have indicated a positive association between NL use and several dietary quality indexes [11,12,13], and between a higher adherence to the Mediterranean diet (MD) [14]. Nevertheless, the scientific knowledge of the associations between NL use and dietary intake remains limited. Thus, given the potential role of NL as a public health tool promoting healthier eating choices, identifying factors for its usage and exploring its association with a healthy dietary pattern such as MD may constitute an important goal of scientific research, especially in order to establish suitable evidence-based approaches for health maintenance and promotion.

For these purposes, young adulthood represents an important transition period in which healthy behavior habits may be acquired and established, thereby positively influencing the health of individuals in later life. Therefore, the aim of this study was to identify determinants of the NL use and explore its association with Mediterranean diet adherence in a cohort of Spanish health university students (DiSA-UMH study) set in a Mediterranean area of Spain.

## 2. Materials and Methods

### 2.1. Study Population and Participants

The DiSA-UMH Project (Dieta, Salud y Antropometría-Universidad Miguel Hernández) is a prospective cohort study of health science university students (72% Medicine, 10% Physiotherapy, 9% Master of Public Heath, 5% Occupational Therapy, and 4% Pharmacy) from Miguel Hernandez University in Sant Joan d’Alacant, Spain. Baseline information was collected for 1204 participants aged 17 to 35 years during the enrolment period from 2006 to 2012. More details about the study rationale and design has been previously described [15]. After excluding the participants with missing data for the main variables and with extreme values for the mean daily energy intake (<500 and >3500 kcal/day for women, <800 and >4000 kcal/day for men), which indicates misreporting [16], 1026 students between 17 and 35 years (288 men and 738 women) were included in the present analysis. All participants provided informed consent, and the Miguel Hernández ethics committees approved the research protocol.

### 2.2. Nutrition Labelling Use Assessment

Participants were asked about NL use with the following question: “Do you usually read the nutrition labelling of packaged foods?” They could choose between two possible responses, “no” or “yes”. This question has been widely used in other epidemiological studies focused on food labelling [17,18]. With this information, the participants were classified into two categories: NL users, when the participants selected “yes”; and NL non-users, when they chose the other response option.

In a subsample of 738 participants of the study, we also collected data about the reasons for using NL or not. NL users were asked “Why do you read the nutrition labelling?” Several optional answers were provided: “For reasons of health or healthy diet”, “To lose and/or control weight”, “For reasons of disease, food allergies, or intolerances”, “Because it is a new brand or product”, “To avoid a specific ingredient or nutrient”, and “Other reasons”. Alternatively, NL non-users were asked the question “Why do not you read the nutrition labelling?” with these possible answers: “Because I do not understand. It is unclear”, “I do not have enough time or I am not interested”, “Because the letters are too small”, “Because the food label is not available”, and “Other reasons”.

### 2.3. Dietary Assessment

Dietary intakes were evaluated using a self-administered semi-quantitative food frequency questionnaire (FFQ) completed at baseline. The FFQ was an adapted version of the questionnaire by Willett et al. [19] and validated for Spanish populations [20,21]. The participants were asked to report how often, on average, they had consumed each food item over the past year. Serving sizes were specified for each food item in the FFQ. The questionnaire offered nine options for frequency of consumption for each food, ranging from never or less than once a month to six or more times/day. Nutrient values for each food in the questionnaire were mainly obtained from food composition tables of the US Department of Agriculture [22] and supplementary Spanish sources [23].

### 2.4. Mediterranean Diet Adherence Assessment

The MD adherence was determined by the relative Mediterranean Diet Score (rMED) [24]. The intake of each component, except for alcohol, was expressed in grams per 1000 kcals and divided into tertiles. A value of 0, 1, and 2 was assigned to the first, second, and third tertiles of intake, respectively, for the six components that presumably fit the MD: fruits (including nuts and seeds), vegetables (excluding potatoes), legumes, fish, olive oil, and cereals (including whole grain). Two components were negatively scored that probably do not fit the MD (lower scoring for the higher intakes): total meat (including processed meat) and dairy products. Owing to the assumed positive effects of moderate alcohol consumption, it was considered as a dichotomous variable using the following ranges: 2 points for moderate consumption (5–25 g/day for women and 10–50 g/day for men) and 0 points for higher or lower consumption.

The overall rMED was calculated for each participant summing the points of the nine components. The resultant scores ranged from 0 points (no adherence) to 18 points (high adherence). The rMED scores were also grouped into three types according to the MD adherence: low (0–6 points), medium (7–10 points), and high (11–18 points).

### 2.5. Covariates

The following information was also collected at baseline: age (years), sex (male, female), smoking status (smoker, non-smoker), self-rated health status (very good/good, fair/poor/very poor), sleep duration (<7, 7–9, >9 h/day), physical activity (not active, moderately active, active/very active), television watching (h/day). More details about self-rated health status, sleep duration, physical activity, and television watching variables are available in previous studies [25,26]. The body mass index (BMI) was calculated by dividing the self-reported weight in kilograms by the squared self-reported height in meters. The self-reported measurements were validated by comparing with weight and height measured in a subsample of the study [27].

### 2.6. Statistical Analysis

Statistical analysis was performed with software R, version 3.0.2 (R Foundation for Statistical). The applied statistical tests were bilateral and significance was established at 0.05. We used the Chi-square or Fisher test for categorical variables and Student’s t-test for continuous variables to compare the sample characteristics between NL users and NL non-users.

To explore the factors associated with NL use, we performed adjusted logistic regression models. The association between MD adherence and NL use was also analyzed by multiple logistic regression analyses. Furthermore, we replicated the same analysis for each component of rMED score.

Models were adjusted for potential confounders based on those factors previously identified in literature and those variables with *p* values < 0.20 in the bivariate analysis. Finally, all models were adjusted for age (years), sex (male, female), total energy intake (kcals/day), sleep duration (<7, 7–9, >9 h/day), physical activity (not active, moderately active, active/very active), and television watching (h/day).

To assess the possible effect of dose-response, linear trend tests were applied for rMED score as continuous variable (low, medium, and high, coded as 1–3).

## 3. Results

The main characteristics of the study population according to the NL use and its predictors are shown in Table 1. Of 1026 participants in the study, 58% used NL, of which 156 (26.2%) were men and 440 (73.8%) women. We observed statistically significant differences (*p* < 0.05) between NL users, who were older, spent less time watching television, and had lower energy intake, and NL non-users. The predictor factors of NL use were women (OR = 1.38, 95% CI: 1.01–1.89), age (OR = 1.08, 95% CI: 1.03–1.13), being physically active/very active (OR = 1.68, 95% CI: 1.17–2.41), and spending less time watching television (OR = 0.84, CI: 0.74–0.95).

Table 2 shows the reasons for using or not using NL examined in a subsample (*n* = 738). The main reasons given by NL users (*n* = 429) were reasons related to health or healthy diet (58.5%) and weight loss and/or control (22.8%), which were, to a large extent, reported by women (67.1% and 81.6%, respectively). Among NL non-users (*n* = 309), the lack of time or interest was by far the most widely given response (77.3%).

The findings from the association between the adherence to MD and NL use are presented in Table 3. Around half of the participants (*n* = 514) had a medium adherence to MD, while the rest of the students were divided almost equally among the low (*n* = 260) and high adherence (*n* = 252) to MD. We noted that the amount of NL users increased proportionally in accordance with higher adherence to MD. Thus, the greatest percentage of NL users was largely concentrated in the group with high adherence to MD (71.4%). We also observed a positive association between NL use and higher adherence to MD. An increase of 2 points in the rMED was significantly related to an increment of 30% in the NL use. Similar results were found when categories of the rMED, i.e., low (0–6), medium (7–10), or high (11–18) score, were compared. Participants with medium or high adherence to MD were significantly more likely to use NL (OR = 1.47, 95% CI: 1.08–2.01 and OR = 2.48, 95% CI: 1.69–3.63, respectively) than those who had low adherence to MD. Furthermore, we observed that the relationship between adherence to MD and NL use showed a significant dose-response association (*p*-trend < 0.001).

Table 4 presents the association between each component of rMED and NL use. NL users were identified as those who had a higher consumption of fruits (OR per 100 g/day = 1.15, 95% CI: 1.08–1.12), vegetables (OR per 100 g/day = 1.22; 95% CI: 1.11–1.34), and fish (OR per 100 g/day = 1.94; 95% CI: 1.38–2.71), and a lower intake of meats (OR per 100 g/day = 0.76; 95% CI: 0.58–0.99), compared with NL non-users.

## 4. Discussion

This study shows that 58% of the students were NL users and were most likely to be women, be older, be physically active/very active, and to spend less time watching television. After adjusting for potential confounding factors, our findings suggest that those university students who had higher adherence to MD use NL greatly and have a larger consumption of fish, vegetables, and fruits and a smaller intake of meats.

In our study, the proportion of NL users was moderate, even considering that the participants were health science students, but similar to that reported in other European studies conducted in the general population, although it was considerably smaller compared with data from the United States [6]. These differences between studies can be, to a certain extent, explained by the fact that the regulation of NL differs substantially among countries and it can contribute to the disparity in the number of NL users. While NL is mandatory in the United States, Canada, or Australia, it is purely voluntary in other countries such as European Community members, except when there is a nutrition or health claim made on foods, suggesting specific beneficial nutrition properties [28]. Additionally, the lower number of NL users generally reported in Europe, where consumers usually show more interest in food safety, freshness, information on origin, and best-before date than nutrition issues [6], may be also a consequence attributed to the lack of awareness regarding the importance of NL as a tool to improve health.

Our results agree with previous studies in that women and older people were more likely to use NL [5,29,30]. This sex difference has been partly interpreted to the fact that many men consider that NL is not a useful tool to help in food choice [4,5]. However, the association between age and NL use is still not clear. Some studies have found a positive link between being older and NL use [11,29], while other reports have led to contrary findings [4,5]. In this study, we observed that the likelihood of using NL increased by 8% per each year of increment in age, which may be explained by the fact that older people can be more concerned about their health and may have higher education regarding nutrition [6].

The positive association between physical activity and NL use has been also shown in previous studies. Satia et al. reported that people who practiced light or moderate physical activity were more likely to use NL [17], although we found a stronger association among the students who reported being physically active or very active. Interestingly, we also observed an inverse association between television watching and NL use. We formerly noted that spending more time watching television was associated with a higher BMI [31], which, according to a previous review [32], could be related to poor eating habits and other sedentary behaviors and several cardiometabolic risk factors and, therefore, could indicate lower NL use. Thus, growing evidence is linking the NL use with those who have a greater interest in healthy eating, show better nutrition knowledge, and present healthier eating patterns [33], which reinforces our findings.

Moreover, our data suggest that NL use may be associated with a healthy diet such as the Mediterranean diet. These results were consistent with that reported by a previous study [34]. These findings contribute to the exploration of new approaches to the role of NL use as a suitable tool to make healthier food choices from a different wider perspective based on dietary patterns beyond the limited viewpoint focused on individual nutrients or foods. In this sense, NL use should be considered a good proxy for identifying dietary patterns.

The association between NL use and a healthier diet such as MD may also indicate that nutrition knowledge could be an important determinant and intermediate factor in this relation [11,34,35]. Recently, nutrition knowledge has also been associated with a higher adherence to MD [35], which may suggest a probable link between healthier dietary patterns and higher health awareness. Thus, we observed that NL users were more likely to have a healthy lifestyle (i.e., being physically active/very active or spending less time watching TV) and to have a low-meat diet rich in fruits, vegetables, and fish. Moreover, when they were asked about the reasons for using NL, they declared more interest in health-related issues. 

This study has several limitations. Firstly, the cross-sectional analysis of the baseline data prevents us from making causal inferences. Furthermore, the fact that the participants were health science students who voluntarily participated in the study might have produced some response bias. In order to minimize bias, we used a validated food frequency questionnaire [20,21] and carried out the validation of self-reported weight and height in a subgroup of the study population [29]. We observed significant associations between NL use and some factors such as sex, age, television watching, physical activity, and adherence to MD, which provide evidence on the determinants of NL use, which is especially lacking in Mediterranean areas. Moreover, we used only one non-validated question on NL use, although it was widely used in other previous epidemiological studies [17,18]. Alternatively, we also asked other questions to explore the reasons for using or not NL, which allowed us to improve the accuracy of NL use.

Our data are far from being able to establish a possible causal link between NL use and higher adherence to MD, but they constitute a suitable rationale for replicating in other samples. Therefore, additional large-scale longitudinal studies are necessary to corroborate our findings and explore other aspects not covered in this study, such as nutritional knowledge, consumer preferences, or participant skills.

## 5. Conclusions

In our study, the prevalence of NL use among young adult people in a Mediterranean area was moderate. Being a woman, older, physically active/very active, and spending less time watching television were statistically significant factors associated with NL use in this population. Moreover, our findings suggested that those university students who were NL users had a higher adherence to MD compared with non-NL users and they were more likely to consume a greater intake of fruits, vegetables, and fish, and a lower intake of meat.

Our approach contributes to exploring the role of NL use as a suitable tool to make healthier food choices from a different wider perspective based on dietary patterns such as the Mediterranean diet, which can also indicate an overall healthy lifestyle. Given a lack of research about the strategies to motivate people in reading NL to improve their food choices and increase their nutritional knowledge, further studies focused on this issue are required.

## Figures and Tables

**Table 1:** Socio-demographic and lifestyle characteristics according to the nutrition labelling use and its predictor factors in participants aged 17–35 years from the DiSA-UMH (Dieta, Salud y Antropometría-Universidad Miguel Hernández) study, Spain (*n* = 1026).

	Nutrition Labelling Use			
	No	Yes	*p*-Value ^1^	OR ^2^ (CI 95%)
	*n* (%)	*n* (%)		
Sex			0.11	
Male	132 (45.8)	156 (54.2)		1
Female	298 (40.4)	440 (59.6)		1.38 (1.01–1.89)
Self-rated health status			0.94	
Very Good/Good	383 (41.9)	530 (58.1)		1
Fair/Poor/Very Poor	47 (41.6)	66 (58.4)		1.14 (0.76–1.74)
BMI (kg/m^2^)				
<25	371 (42.0)	513 (58.0)	0.93	1
25.0–29.9	49 (42.2)	67 (57.8)		0.96 (0.64–1.46)
≥30	10 (38.5)	16 (61.5)		1.18 (0.51–2.73)
Smoking status			0.89	
No	292 (42.1)	402 (57.9)		1
Yes	138 (41.6)	194 (58.4)		0.98 (0.74–1.30)
Sleep duration (h/day)			0.09	
<7	31 (36.5)	54 (63.5)		1.25 (0.77–2.01)
7–9	367 (41.6)	515 (58.4)		1
>9	32 (54.2)	27 (45.8)		0.66 (0.38–1.14)
Physical activity			0.10	
Not active	252 (44.8)	310 (55.2)		1
Moderately active	99 (38.8)	156 (61.2)		1.34 (0.98–1.83)
Active/very active	79 (37.8)	130 (62.2)		1.68 (1.17–2.41)
	**Mean (SD)**	**Mean (SD)**	***p*-Value ^1^**	**OR ^2^ (CI 95%)**
Age (years)	22.6 (2.6)	23.2 (3.3)	<0.05	1.08 (1.03–1.13)
TV watching (h/day)	1.5 (1.0)	1.3 (1.0)	<0.05	0.84 (0.74–0.95)
Energy intake (kcals/day)	2125 (621)	2004 (606)	<0.05	0.99 (0.99–1.00)
Alcohol intake (g/day)	4.5 (7.7)	4.4 (6.7)	0.86	0.99 (0.97–1.01)

Abbreviations: BMI, Body Mass Index; SD, standard deviation; CI: confidence interval; TV, television. ^1^ *p*-value from Chi-square test (categorical variables) and t-student test (continuous variables); ^2^ OR: Odds ratios were adjusted by all variables in the table.

**Table 2:** Reasons for using or not using nutrition labelling given by students from the DiSA-UMH study, Spain (*n* = 738). NL, nutrition labelling.

	Total	Men	Women
	*n* (%)	*n* (%)	*n* (%)
Reasons for using NL	429 (100.0)	141 (32.9)	288 (67.1)
Health or healthy diet	251 (58.5)	90 (35.9)	161 (64.1)
To lose and/or control weight	98 (22.8)	18 (18.4)	92 (81.6)
Disease, food allergies, or intolerances	8 (1.9)	4 (50.0)	4 (50.0)
New brand or product	35 (8.2)	8 (22.9)	30 (77.1)
To avoid a specific nutrient or ingredient	6 (1.4)	3 (50.0)	3 (50.0)
Other reasons	31 (7.2)	18 (58.1)	16 (41.9)
Reasons for not using NL	309 (100.0)	92 (29.8)	217 (70.2)
I do not understand. It is unclear	23 (7.4)	8 (34.8)	15 (65.2)
I do not have enough time or I am not interested	239 (77.3)	71 (29.7)	168 (70.3)
The letters are too small	15 (4.8)	5 (33.3)	10 (66.7)
Food label is not available	12 (3.9)	4 (33.3)	8 (66.7)
Other reasons	20 (6.5)	4 (20.0)	16 (80.0)

**Table 3:** Relationship between adherence to the Mediterranean Diet (rMED) and nutrition labelling use in participants aged 17–35 from the DiSA-UMH study, Spain (*n* = 1026).

	Total (*n*)	NL Use (%)	OR (CI 95%) ^1^	*p*-Trend
rMED (0–18)				
Per 2-point increase ^2^	1026	58.0	1.30 (1.18–1.43)	**-**
Per category increase ^3^				<0.001
Low (0–6)	260	45.8	1.00	**-**
Medium (7–10)	514	57.8	1.47 (1.08–2.01)	**-**
High (11–18)	252	71.4	2.48 (1.69–3.63)	**-**

^1^ Odds ratios were adjusted for sex (male, female), age (years), sleep duration (<7, 7–9, >9), physical activity (not active, moderately active, active/very active), TV watching (h/day), energy intake (kcals/day). ^2^ Relative Mediterranean Diet Score (rMED) included as a continuous variable in the model. ^3^ Relative Mediterranean Diet Score (rMED) included in categories in the model. OR: Odds ratio; CI: confidence interval. -: Not applicable

**Table 4:** Relationship between food components of Relative Mediterranean Diet Score (rMED) and nutrition labelling use in participants aged 17–35 years from the DiSA-UMH study, Spain (*n* = 1026).

	Nutrition Labeling Use
Daily Intake (g/Day)	OR ^1^ (95% CI)
Fruits (100 g/day)	1.15 (1.08–1.12)
Vegetables (100 g/day)	1.22 (1.11–1.34)
Legumes (10 g/day)	1.03 (0.98–1.08)
Cereals (10 g/day)	1.12 (0.95–1.33)
Fish (100 g/day)	1.94 (1.38–2.71)
Olive oil (10 g/day)	1.01 (0.93–1.11)
Dairy products (100 g/day)	1.05 (0.99–1.12)
Meats (100 g/day)	0.76 (0.58–0.99)
Alcohol (10 g/day)	0.95 (0.79–1.13)

^1^ Odds ratios were adjusted for sex (males, female), age (years), sleep duration (<7, 7–9, >9), physical activity (not active, moderately active, active/very active), TV watching (h/day), energy intake (kcal/day); OR: Odds ratio; CI: confidence interval.
